#  Sirenomelia: two case reports 

**DOI:** 10.1186/s13256-021-02699-4

**Published:** 2021-04-26

**Authors:** Asiyeh Shojaee, Firooze Ronnasian, Mahdiyeh Behnam, Mansoor Salehi

**Affiliations:** 1 Division of Physiology, Department of Basic Sciences, Faculty of Veterinary Medicine, Ferdowsi University of Mashhad, Mashhad, Iran; 2 Medical Genetics Center of Genome, No 54319, Genome Building, Mohtasham St., Isfahan, Iran; 3 Cellular, Molecular and Genetic Research Center, Isfahan University of Medical Sciences, Isfahan, Iran

**Keywords:** Sirenomelia, Mermaid syndrome, Caudal regression

## Abstract

**Background:**

Sirenomelia, also called mermaid syndrome, is a rare lethal multi-system congenital deformity with an incidence of one in 60,000–70,000 pregnancies. Sirenomelia is mainly characterized by the fusion of lower limbs and is widely associated with severe urogenital and gastrointestinal malformations. The presence of a single umbilical artery derived from the vitelline artery is the main anatomical feature distinguishing sirenomelia from caudal regression syndrome. First-trimester diagnosis of this disorder and induced abortion may be the safest medical option. In this report, two cases of sirenomelia that occurred in an white family will be discussed.

**Case presentation:**

We report two white cases of sirenomelia occurring in a 31-year-old multigravid pregnant woman. In the first pregnancy (18 weeks of gestation) abortion was performed, but in the third pregnancy (32 weeks) the stillborn baby was delivered by spontaneous vaginal birth. In the second and fourth pregnancies, however, she gave birth to normal babies. Three-dimensional ultrasound imaging showed fusion of the lower limbs. Neither she nor any member of her family had a history of diabetes. In terms of other risk factors, she had no history of exposure to teratogenic agents during her pregnancy. Also, her marriage was non-consanguineous.

**Conclusion:**

This report suggests the existence of a genetic background in this mother with a Mendelian inheritance pattern of 50% second-generation incidence in her offspring.

## Background

Sirenomelia is a rare congenital anomaly in which the most noticeable feature is fusion of the lower limbs, resulting in the appearance of a mermaid's tail, and thus the name mermaid syndrome. This syndrome is characterized by severe urogenital abnormalities and the presence of a singular umbilical cord blood vessel. Sirenomelia or sirenomelia sequence was originally described by Rocheus in 1542 [1]. All the anomalies of mermaid syndrome as the most severe form of caudal regression syndrome were defined by Duhamel in 1961 [2].

Sirenomelia is a lethal disorder with a rare chance of live birth, varying from 0.1:10,000 to 0.47:10,000 based on different reports [3]. Because of the common similarity of this defect to caudal dysgenesis (CD) and VACTERL (vertebral defects, anal atresia, cardiac abnormalities, tracheoesophageal fistula with esophageal atresia, renal and limb abnormalities), the distinction between sirenomelia and similar defects is challenging. Thus there is still debate about whether sirenomelia is a separate entity or is another class of the aforementioned multisystemic malformations [4].

**Fig. 1:**
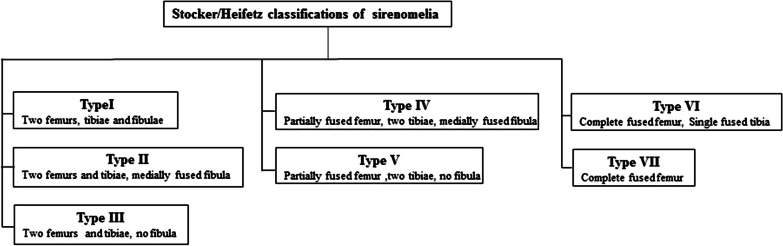
Overview of the classification of sirenomelia

Sirenomelia can be classified into several categories based on the wide variety of limb malformation phenotypes. Saint-Hilaire and Forster classified sirenomelia based on the number of feet. The other widely used classification is the Stocker and Heifetz method, which has seven types (I–VII) and is based on the presence or absence of the femur, tibia, and fibula (Fig. 1) [2, 5, 6]. Other, less commonly used classifications include the Kjaer method based on the iliac-sacral distance (ISD) [6].

The etiology of sirenomelia remains unclear, and most cases are sporadic, with very little chance of incidence in subsequent pregnancies. However, the case of this study is unique as it reports two occurrences of sirenomelia in one family.

## Case presentation

**Fig. 2:**
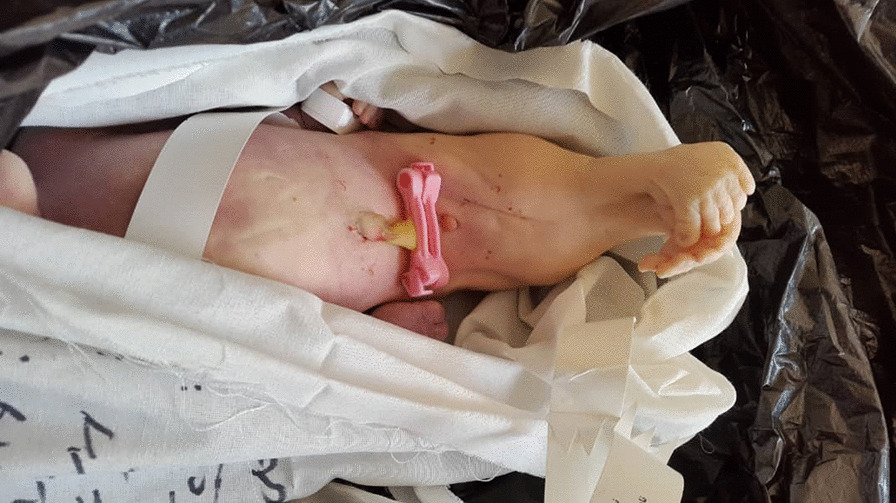
Stillborn baby with sirenomelia

A multigravid 31-year-old white woman and her 35-year-old white husband were admitted for pre-pregnancy consulting for their fourth pregnancy. In their first pregnancy, no fetal movement was detected by sonography at the 18th gestational week. Subsequently, three-dimensional ultrasound imaging showed a fetus with cystic kidneys and fusion of the lower limbs, so the pregnancy was terminated by induced abortion. The woman later gave birth to a normal baby at gravida 2, but at 32 weeks of her gravida 3, vaginal delivery following intrauterine fetal death (IUFD) occurred. The physical examination of the stillborn baby showed typical Potter facies and normal upper part of the body with fused lower limbs and two feet. The two feet were fused, and the femur and tibiae with four toes were discriminable. The external genitalia and anal opening were absent, as shown in Fig. 2. Although the exact type of anomaly cannot be identified in the absence of radiographic findings, it may be classified as type II or III based on the Stocker and Heifetz classification, since the fusion somewhat affects superficial tissue. There was no report of diabetes mellitus (DM) or other anomalies in the mother’s medical history or familial medical history. Moreover, their marriage was non-consanguineous, and the mother had not had exposure to any intrinsic or extrinsic factors (such as teratogenic drug intake) associated with sirenomelia during her pregnancy. Further investigation could not be conducted because the parents refused autopsy.

Eventually, the follow-up to her fourth pregnancy indicated that she gave birth to a normal baby.

## Discussion and conclusions

### Pathogenetic hypotheses of sirenomelia

Although the etiology of sirenomelia is still unclear, there are two chief pathogenetic hypotheses. One of these is defective blastogenesis, which involves deficient mesodermal generation and subsequent impairment in the formation of caudal structures. Based on this theory, sirenomelia could be considered a severe form of CD [2].

Another hypothesis for its etiology is an abnormal vasculature pattern. In most cases, fetuses with sirenomelia exhibit a single umbilical artery (SUA) with an abnormal origin, derived from the vitelline artery. A normal fetus, on the other hand, has two umbilical arteries [7] with a normal origin and no involvement of other malformations [8]. Below the origin of the SUA, the aorta becomes abnormally narrow and lacks a considerable number of branches that normally supply the kidneys, large intestine, and genitalia. The blood is shunted away from the absent or hypoplastic arteries into the SUA, which diverts blood flow to the placenta, causing defects in circulation and nutrient supply to the lower limbs, leading to their arrested development [2].

### Risk factors

Risk factors such as maternal diabetes, heavy metal exposure for sirenomelia, and CD are well established in both experimental models and humans, although only 0.5–3.7% of reported cases had diabetic mothers [9]. The frequency of sirenomelia in monozygotic twins was found to be 150 times the rate observed in dizygotic twin or singleton pregnancies [10]. Interestingly, Orioli *et al*. reported that the risk of sirenomelia increases in cases with mothers younger than 20 years of age. Nevertheless, there is a strong relationship between maternal diabetes or twin pregnancy and higher maternal age in cases of sirenomelia [11]. An association between sirenomelia and assisted reproductive technologies, namely intracytoplasmic sperm injection (ICSI), has also been described [12].

### Teratogenic factors

Exposure to extrinsic elements such as teratogenic agents can lead to sirenomelia. In this regard, some studies have found a relationship between sirenomelia and certain teratogenic drugs such as methylergonovine maleate [13], phenobarbital [14], antibiotic trimethoprim [15], and misoprostol [11], although these findings may be coincidental. In experimental studies, successful induction of sirenomelia by administration of cadmium and lead in the golden hamster was reported [16]. In another report, sirenomelia was induced in chicken embryo by irradiation exposure on the axial portion of the caudal region and in hamsters by retinoic acid (RA) treatment or irradiation exposure [6].

### Genetic aspect of sirenomelia

Unlike in humans, the genetic basis in the animal model is well established. A sirenomelia-like phenotype in animal models has been illustrated by genetic modifications through induction of loss-of-function (LOF) mutations in bone morphogenetic protein (Bmp) or gain-of-function (GOF) mutations in RA signaling. The genetic disruption of the key enzyme that degrades RA, Cyp26a1, reportedly induced sirenomelia in mice [17]. The administration of RA to pregnant rats resulted in abnormal development of the umbilical and vitelline arteries [6]. Therefore, it has been claimed that the involvement of RA signaling in sirenomelia may have genetic or epigenetic roots.

Bmp signaling plays a vital role in the remodeling of the vasculature [18]. There is cross-talk between the RA and BMP pathways, as RA levels are increased by decreasing Bmp signaling [19].

Unlike mutant mice with sirenomelia, such as Bmp7 on 20q13.31, Bmp4 on 14q22.2, and Cyp26B1 on 2p13.3, very few chromosomal conditions have been reported in humans. Recently, a *de novo* balanced translocation involving chromosome 16 and triploid mosaicism was reported [20, 21].

### Familial occurrence

Orioli *et al*. [11] reported no familial recurrence of sirenomelia in their study; however, true recurrence of sirenomelia was reported at least in one study [6, 22]. In most familial cases, sirenomelia occurs along with CD-related anomalies, and most of these are sporadic cases [2, 6]. In our cases, the recurrence of sirenomelia occurred in one family. This report suggests a genetic basis for sirenomelia with a Mendelian inheritance pattern of 50% second-generation incidence in offspring.

### Diagnosis

Diagnosis of sirenomelia is more feasible in the first trimester than later in pregnancy because there is still a sufficient amount of amniotic fluid that is less affected by fetal urinary production [23]. Oligohydramnios in the second half of pregnancy makes diagnosis more difficult due to the presence of urogenital abnormalities.

Ultrasound examination is the gold standard method for sirenomelia diagnosis. Color and power Doppler ultrasound is helpful in the diagnosis of the single umbilical artery, especially in oligohydramnios cases [7]. A first-trimester ultrasound test should be done to minimize the physical and psychological trauma related to the termination of pregnancy at longer gestational periods.

## Conclusion

The roles of maternal diabetes, exposure to heavy metals, and RA have been acknowledged in the incidence of sirenomelia, and have been described as important environmental risk factors. Given its sporadic nature in humans, it is possible that sirenomelia occurs due to an autosomal-dominant genetic background; however, it is more likely that a combination of genetic and environmental components are responsible. The limitations of this study including a lack of ultrasound images and postnatal investigations prevent us from reaching an ultimate conclusion on the disease etiology, but the pedigree of this family could add value to the literature.

## Data Availability

The sources for the information discussed in this report can be obtained from the papers cited in the references.
